# Optofluidic Technology for Water Quality Monitoring

**DOI:** 10.3390/mi9040158

**Published:** 2018-04-01

**Authors:** Ning Wang, Ting Dai, Lei Lei

**Affiliations:** 1 National Engineering Laboratory for Fiber Optic Sensing Technology, Wuhan University of Technology, Wuhan 430070, China; tingdai@whut.edu.cn; 2 Shenzhen MacRitchie Technology Co., Ltd., Shen Zhen 518101, China

**Keywords:** optofluidic device, water quality, detection

## Abstract

Water quality-related incidents are attracting attention globally as they cause serious diseases and even threaten human lives. The current detection and monitoring methods are inadequate because of their long operation time, high cost, and complex process. In this context, there is an increasing demand for low-cost, multiparameter, real-time, and continuous-monitoring methods at a higher temporal and spatial resolution. Optofluidic water quality sensors have great potential to satisfy this requirement due to their distinctive features including high throughput, small footprint, and low power consumption. This paper reviews the current development of these sensors for heavy metal, organic, and microbial pollution monitoring, which will breed new research ideas and broaden their applications.

## 1. Introduction

Water pollution has become one of the most pressing environmental problems in the world today [1,2,3,4]. Water research, especially water pollution analysis, has drawn attention from the general public. The analytical methods must be sensitive, accurate, high-speed, and automatic. Most detection technologies in modern analytical chemistry have been used in water pollution analysis, such as plasma emission spectrometry [5], atomic fluorescence spectrometry [6], gas chromatography–mass spectrometry [7], and high-performance liquid chromatography [8]. While researchers are still continuing to develop large-scale, sophisticated monitoring systems, there is an emerging trend towards portable, automated, continuous, simple, and fast detection devices [9,10,11,12,13].

Traditional methods to monitor water pollutants normally need to collect samples from a reservoir or watershed and then send them to laboratories for testing and a final analysis report. Long operation time may lead to inaccurate results because the water samples may undergo chemical, biological, and physical reactions during this period. Although certain on-site or in-line instruments are available to monitor pH, DO (dissolved oxygen), ORP (oxidation reduction potential), and various cations and anions of water in the market, their bulk size and high energy consumption limit their application. In addition, certain lab-based detection methods are difficult to package into an on-site system, especially for microbial monitoring, because of the operational complexity and requirement of an ultra-clean operating environment [14,15,16,17,18,19,20,21].

Microfluidics, also known as lab-on-a-chip or biochip, refers to techniques to control, operate, and detect fluids at microscopic dimensions. It can realize the basic functions of chemical and biological laboratories on a chip, with the goals of miniaturizing, automating, and integrating multifunction from processing to testing samples through the intersection of chemistry, micromachining, computers, electronics, materials science, and biology [22,23]. Optofluidics, defined as a fusion of optics and microfluidics, has recently become a hot research topic due to its advantages such as integration, miniaturization, and high precision, and it shows high potential in applications of biomedical and environmental sensing. 

Although a few attempts to commercialize the related technology for the real-world application have been reported, such as eventlab from optiqua and parasitometer from Water Optics Technology, optofluidic devices for water pollution monitoring are mostly used in laboratories. At present, there is a serious lack of systematic reviews on its applications in the field. In this review, recent progress on optofluidic devices is reported with a focus on the various methods that are used to detect chemical, microbiological, and other ecological pollutants. Moreover, we highlight several commercial products and discuss their potential applications to water research and environment science.

## 2. Optofluidics for Online Water Quality Monitoring

Optofluidics, a new interdisciplinary combination of microfludics and optics, integrates fluid and optical elements, making it feasible to create innovative sensors with enhanced selectivity, adjustability, and compactness.

Optofluidic devices integrate microfluidic and optical components onto one chip and tie them together with high interaction efficiency between light and fluids, which is utilized for sensing applications [24]. Furthermore, using a continuous microchannel network to drive microfluids in and out of the handling system makes the optofluidic devices very attractive for environmental and biological threat detection, especially for continuous online measurement of trace amounts [25]. During the last two decades, many researchers have demonstrated numerous fundamental optofluidic elements such as optofluidic waveguides, including liquid core Anti-Resonant Reflecting Optical Waveguides (ARROWs) [26,27,28], Photonics Crystal Waveguides [26], Slot Waveguides [26,29,30], Liquid-Liquid Waveguides [31], Jet Waveguides [32], etc. and optofluidic devices including on-chip spectroscopy, an interferometer, and a resonator. Considering their potential application for detecting diverse chemical and biological components, it is important to realize water quality monitoring using compact, low-cost, low energy consumption and in-line optofluidic devices.

Because of the large-scale use of pesticides, heavy metal pollutants, and airborne metallic pollutants, aquatic ecosystems are threatened on a global scale [33]. The current water quality testing methods can be placed in three categories: (a) artificial fixed-point sampling and then laboratory off-line analysis; (b) portable instruments for on-site manual sampling test; and (c) continuous sampling from the fixed test site and automatic online analysis in real time. The manual fixed-point sampling has low efficiency, high cost, and is difficult to maintain. A portable instrument for on-site manual sampling is usually a single instrument to measure parameters with a limited range; often it has low efficiency and on-site calibration problems. The last method has high operating costs and can only obtain water quality testing results at a fixed site. 

Optofluidic devices are good candidates for online continuous water quality monitoring because they can be integrated into different application fields, have reasonably low manufacturing cost and small chemical/energy consumption, which will minimize the hardware investment and operational costs.

According to the WHO drinking water safety guidelines [34], chemical and microbial pollutants are the main risks to water quality safety. In the following paragraphs, we present recent progress in research on chemical and microbial pollutants detection using optofluidic devices.

## 3. Chemical Pollutants Detection

The sources of chemical constituents in drinking water can be divided into the following three kinds of categories: natural origin from rocks/clays, and the geological environment and climate. Moreover, waste from factories and residences, agricultural processes, water governance, and substances relating to pesticides were also included [34]. More than 100 different chemical components were proven to be harmful for people’s health; they can be classified into inorganic anions, heavy metals ions, and organic pollutants.

### 3.1. Inorganic Anions

#### 3.1.1. Optical Absorption Change by Chemical Reaction

Optofluidic systems for nutrient determination are mostly based on spectrophotometric detection, using microfabrication techniques to integrate various optoelectronic components. For example, an optofluidic chip is integrated with a data transmitting device for phosphate detection in water [33]. The system can be equipped in different positions to conduct a full range of on-line searching for phosphate pollution in the target area with a detection limit of at most 0.3 mg/L. A Fabry–Pérot resonator, which enhances absorption, is used to detect marine phosphates. The optofluidic system can detect phosphate in real time and is of great significance for the detection and prediction of harmful algal blooms in the marine environment [35].

**Figure 1:**
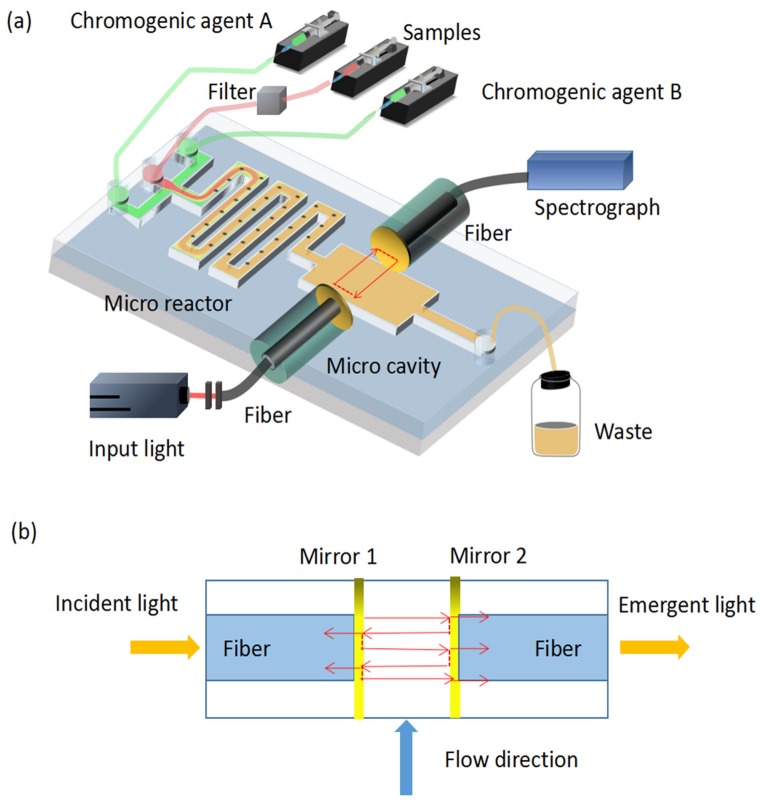
An optofluidic device based on the Fabry–Pérot resonator used to detect marine phosphate. (**a**) A 3D schematic of the setup. The microchannel area is 250 μm of width; the F–P cavity is 300 μm of length. (**b**) The microcavity is composed of two pairs of gold-coated optical fiber facets [35].

Zhu et al. [35] designed a combination technology of optofluidics with microscale resonators for the detection of phosphate. As shown in Figure 1a, the system was composed of a Fabry–Pérot microcavity with two parallel fiber facets coated with an Au film (Figure 1b) and a microchannel with a width of 250 µm. The water-soluble components, which were controlled by the flow rates, consisted of a phosphate solution, ascorbic acid solution, and a mixture of 12% ammonium molybdate solution, 80% concentrated sulfuric acid, and 8% antimony potassium tartrate solution. Compared to the traditional methods, the optofluidic device could shorten the detection time from 10 min to 6 s and increase the detection limit to 100 μmol/L.

**Figure 2:**
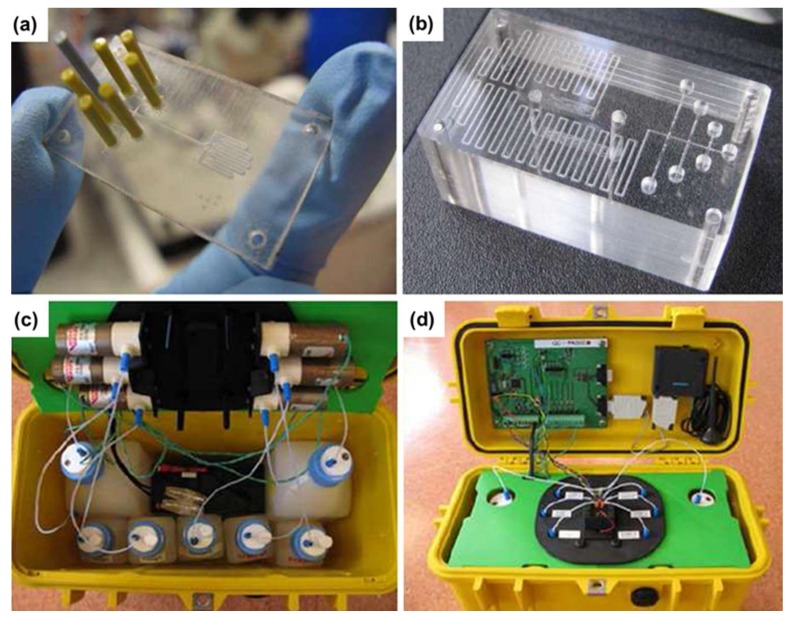
The optofluidic chips and assembled system used for phosphate detection. A high-sensitivity absorbance optical detection module is integrated with the optofluidic chip to monitor the colorimetric reaction of 20 µL volume reagent and sample. (**a**) The physical photo of microfluidic chip is described in this section. It shows a curved channel on the right side and six inlets on the left side with one outlet [36]. (**b**) Another chip redesigned to mix the sample and reagent, and the area used to detect is substituted with a cylindrical optical cuvette [36]; (**c**) an integrated device with bottles and battery [37]; (**d**) top board and cover showing electronic board and GSM modulator [37].

In addition, an optofluidic device for performing colorimetric measurement is presented in Figure 2a [36]. It allows a colorimetric reaction using a very small volume of sample and reagent (i.e., 20 µL), which are mixed in the T-mixer and connected by a serpentine channel where the interaction solution is provided to the emitter and photodetector for absorbance testing. Three inlets were designed on both sides of the T-mixer. The standard phosphate solutions and the water sample for calibration were introduced from one side. The reagent and two channels are for cleaning with deionized water (DI water) on the other side. In the microchannel, ammonium molybdate, (NH_4_)_6_Mo_7_O_24_·H_2_O, is reacted with ammonium metavanadate, NH_4_VO_3_, under acidic conditions. After being fully mixed and reacted, the vanadomolybdophosphoric acid complex, (NH_4_)_3_PO_4_NH_4_VO_3_·16MoO_3_, was generated in the resulting solution, which presented a distinct yellow color, and therefore was accompanied by the strong absorbance below 400 nm. Also, the long-term reliability of the device was demonstrated in this work. From the experimental results, it has been proven to have a limit of detection (LOD) of 0.2 mg/L and a dynamic linear range of 0–50 mg/L. Due to the limited storage capacity, the optofluidic device could allow for >11,000 measurements.

Figure 2c,d show the fully assembled system [37]. With a Pelicase enclosure, five storage bottles and two waste bottles were fixed at the bottom. The black box between the waste bottles was the battery. Six solenoid pumps were fixed on the flipped top plate, as shown in Figure 2a. When we close the upper and lower plates, all the inlets and outlets can be accessed and connected with the microfluidic chip automatically. Moreover, the temperature sensor, LED, and photodiode were also configured on the top cover.

#### 3.1.2. Fluorescence Quenching of Gold Nanoparticles in Water

For the detection of cyanide in tap water, drinking water, lake water, seawater, and industrial sewage, fluorescence quenching detection is developed using an optofluidic device. The integrated optofluidic device measures the fluorescence and colorimetric properties of gold nanoparticles mixed with the target water sample [38]. In these studies, the surface of Au NPs is modified by fluorescein isothlocyanate (fluorescent dye), which results in the fluorescence being quenched. Cyanide reacts with the Au NPs, forming a soluble gold/cyanide complex. The dye may then restore the fluorescence intensity. To stabilize Au NPs against high ionic strength, polysorbate 20 was used. When cyanide is present at the concentration larger than 150 µM, Au NPs’ aggregation appears. In this way, the fluorescence could be detected at low concentrations of cyanide (LOQ of 10 µM). Thus, the colorimetric method is recommended for high concentration conditions (>150 µM).

The method described above has great sensitivity to cyanide compared to other common ions present in aqueous samples. The choice of detection method, fluorescence or colorimetric, depends on the amount of cyanide in the sample. In some cases with industrial water, the colorimetric method is more desirable.

### 3.2. Heavy Metal

#### 3.2.1. Bioluminescence Inhibition of Specific Bacteria

**Figure 3:**
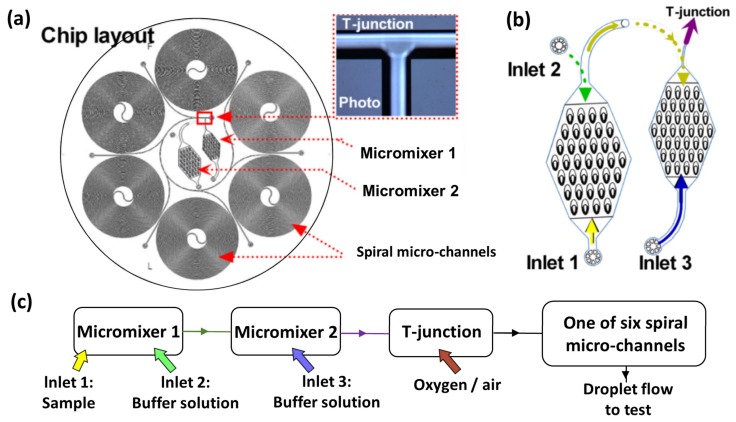
The bioluminescent cell-based optofluidic device for toxicant detection in water environment. *V. fischeri* is used as the toxicity indicator based on inhibition of luminescence; the device is designed to work continuously and shows high potential for home usage. (**a**) The optofluidic chip is composed of two counter-flow micromixers, a T-junction droplet generator, and six spiral microchannels; (**b**) two counter-flow micromixers and the flowing direction and mixing process; (**c**) the function of the different microstructures on the optofluidic chip [39].

The method of luminescent bacteria testing is mainly to suppress the photoexcitation of *Vibrio fischeri* cells in response to the toxic effects caused by heavy metal ions. *Vibrio fischeri* luminesces by generating luciferase, which catalyzes the oxidation of long-chain fatty aldehydes and reduces flavin mononucleotides, with free energy being released with luminescent of excited wavelength 490 nm. The traditional inhibition test is usually done by adding a volume of a luminescent bacterial suspension to the cuvette. The criterion for the test judgment was the attenuation of luminous intensity measured after 15 min and 30 min. In an optofluidic system, by processing in parallel the samples and standard liquid and measuring the luminescent intensity changes, the inhibition rate could be determined conveniently and rapidly. In Zhao’s work [39], a novel optofluidic system based on the above principles and one-off toxicity assessments in ISO11384 was designed and characterized, as shown in Figure 3. The system worked via the following steps. First, the sample and luminescent bacterial suspension were continuously pumped into the micromixer at the same flow rate. After that, the mixture passed through a spiral microchannel to the viewing chamber. The optical measurement usually takes 20–30 min. Under fair control conditions of the Lab-on-a-Chip (LOC) system, the inhibition rate based on the luminescent intensity in the parallel microchamber could be calculated by observing and comparing the two steady states.

#### 3.2.2. Color Change by Immunological Reaction

To achieve reliable observations of the bioluminescence inhibition, an observation chamber integrated with a highly sensitive photomultiplier tube (PMT) was designed in the optofluidic chip and its volume was optimized together with the droplet flow rate. Heavy metal ions (Hg^2+^, Cd^2+^ and Pb^2+^) in rivers, lakes, mineral and tap water could be detected and characterized by the antibody/antigen reaction in an optofluidic system. As reported by Zhou’s group [40], a nitrocellulose membrane was covered separately by goat anti-mouse IgG antibody and the coating antigens Hg^2+^-ITCBE-BSA, Cd^2+^-ITCBE-BSA and Pb^2+^-ITCBE-BSA(test-lines). Hg^2+^, Cd^2+^, and Pb^2+^ ions in water samples bind with the mAb/particle conjugates (specific mAb-labeled Au NPs), preventing their migration and capture by immobilized Hg^2+^-ITCBE-BSA, Cd^2+^-ITCBE-BSA, and Pb^2+^-ITCBE-BSA. By using grayscale densitometry, the results show that the change of color density was inversely proportional to the concentration of metals.

This method allows the rapid (<10 min) and detection of multiple metal ions simultaneously with a high level of selectivity. The LODs for Hg^2+^, Cd^2+^, and Pb^2+^ were 8, 6, and 6 nM, respectively.

#### 3.2.3. Absorbance Change of Nanoparticles

Hg^2+^ ions can oxidize Ag nanoparticles (stabilized by sodium citrate) to Ag^+^, causing the disintegration of nanoparticles. At the same time, Hg adsorbs on the Ag NPs, which results in the displacement of citrate molecules from the Ag NP surface and a decreasing negative charge of the particles. This leads to the enlargement of Ag NPs and a change in absorbance.

In an optofluidic device for Hg^2+^ detection [41], functionalized nanoparticles were fully mixed with the water sample in the microchannels and the absorbance of the solution was measured via a UV-Vis spectrophotometer. The measured range of Hg^2+^ concentration was 0.5–7 ppm, with precision RSDs of 3.24–4.53. The presence of Cu^2+^ ions enhances the sensitivity of Hg^2+^ detection: the LOD improves from 0.06 to 0.008 ppm.

### 3.3. Organic Pollutants

#### 3.3.1 Micro-Ring Resonating Status Change by Immunological Reaction

Compared to inorganic pollutants in water, organic pollutants are more abundant. They affect ecosystems in a toxic and water-soluble manner and endanger human health. Therefore, the quantity of organic pollutants is an extremely important indicator of water pollution status. Due to their low content, these pollutants need to be pretreated in the early stage. The advantages of microfluidics include the ability to integrate the pretreatment and afterward detection, high extraction/enrichment efficiency, etc.

**Figure 4:**
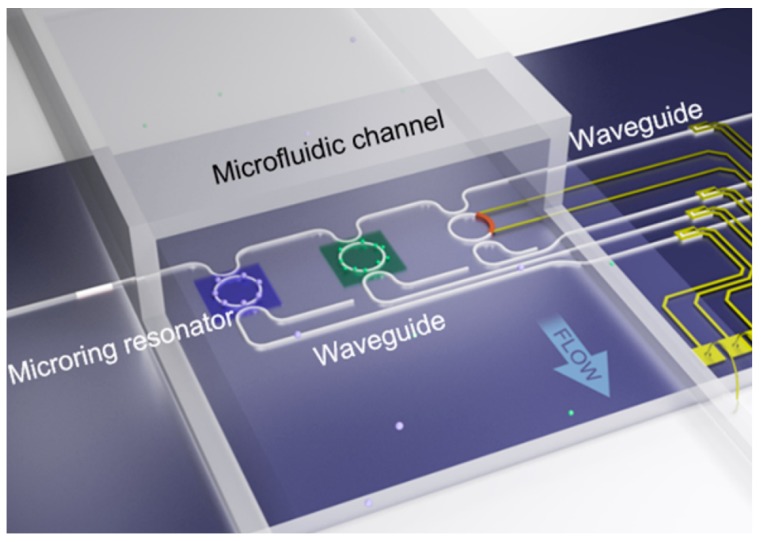
The optofluidic chip used to detect 2, 4-Dichlorophenoxyacetic [42].

The optofluidic chip [42] shown in Figure 4 was used for sensitive monitoring of 2,4-Dichlorophenoxyacetic acid from different sources. Due to its excellent selection of antibodies, it has extremely high selectivity for 2,4-Dichlorophenoxyacetic acid. The optofluidic chip is fabrication by covalent immobilization of 2,4-D-bovine serum albumin (2,4-D-BSA) conjugate to an integrated microring resonator. It has a limit of detection (LOD) of 4.5 pg/mL and a quantitative range of 15–105 pg/mL. Its LOD was several orders of magnitude lower than conventional assays such as commercial enzyme linked immunosorbent assay (ELISA) kits, electrochemical impedance, fluorescence-labeled immunosensors, and APRs. More importantly, it requires a lower solution volume (~20 µL) than traditional ELISA methods (>100 µL).

#### 3.3.2. Fluorescence Intensity Change by Immunological Reaction

Compared to traditional techniques such as ELISA, HPLC-UV, and invertebrate bioassay, an automated online optical biosensing system is rapid, sensitive, and high-frequency online-monitoring system for microcystin detection, and it is based on the principle of the total internal reflection fluorescence and flow indirect immunoassay. In Shi’s research work [43], an innovative automated online optical biosensing system (AOBS) was designed to rapidly detect microcystin-LR (MC-LR), one of the most toxic cyanotoxins in water. MC-LR and anti-MC-LR-mAb within a certain concentration were premixed and pumped onto the chip surface. The laser was opened, at the same time exciting the surface-bound fluorescence and using an optical detector to record. The concentration of MC-LR, for which the LOD is 0.09 µg/L, ranges from 0.2 to 4 μg/L. The system has abilities of on-line detection and early warning response to water pollution, which has been successfully applied in Lake Tai in China. This novel system design was demonstrated with potential for detecting cyanobacteria and the reduction of pollution in fresh water, surface water, and drinking water. 

Zhou et al. demonstrated that microfluidic systems also have important applications in the testing of chemical feedstocks [44]. In their study, an evanescent wave-excited immunological biosensor was designed to detect bisphenol A (BPA) with the detection limits of 0.03 mg/L. It is a portable device that only takes 20 min per assay cycle and can perform more than 300 assay cycles. This technique has low LOD, high sensitivity, and excellent selectivity in a real water environmental matrix. Due to the reconfiguration of the planar optical waveguide microchip, it is possible to have high testing frequency for water pollution detection.

## 4. Microbial Pollutants Detection

Microbial pollutants such as pathogenic bacteria, viruses, and parasites in water samples are very common [34]. Their typical size ranges from tens of nanometer (virus) to tens of micrometer (protozoa), which is far smaller than the eye can see. The current lab-based test methods are normally time-consuming (24 h to 7 d) and involve complicated procedures, normally including water sample filtration, microbial purification, culture, and observation by microscopic methods. Flow cytometry is an alternative method for their determination. However, its equipment is expensive, bulky, and requires special operation, and therefore it is not suitable for on-site and continuous monitoring.

Based on their size, the microorganisms in water bodies belong to the scope of particulate organic carbon. Their species groups can reflect the ecological characteristics of water bodies and the pollution status, which is the routine monitoring index in water ecological surveys. The emergence of an optofluidic device based on sheath flow fluid control makes the integration, miniaturization, automation, and portability of the instrument possible. In addition, the combination of nucleic acid or immunological bioassay methods and immunomagnetic separation technology for the detection of waterborne pathogens is of great significance.

### 4.1. Microdroplet Scattering Change by Bio-Reaction

Traditionally, the marking method was used for bacteriophage detection, which has the disadvantages of complex, time-consuming steps and a low recovery rate. In Yu’s research work [45], a label-free method was employed to detect phages, here named droplet optofluidic imaging. Phage infection reaction assays used droplets containing host cells as a carrier due to the higher proportion of phages and host cells in the droplets. Optofluidic imaging relied on the variety of the effective refractive index of the growth rate of infected host cells in the droplet, presents high sensitivity, and can even detect a single *E. coli* cell.

**Figure 5:**
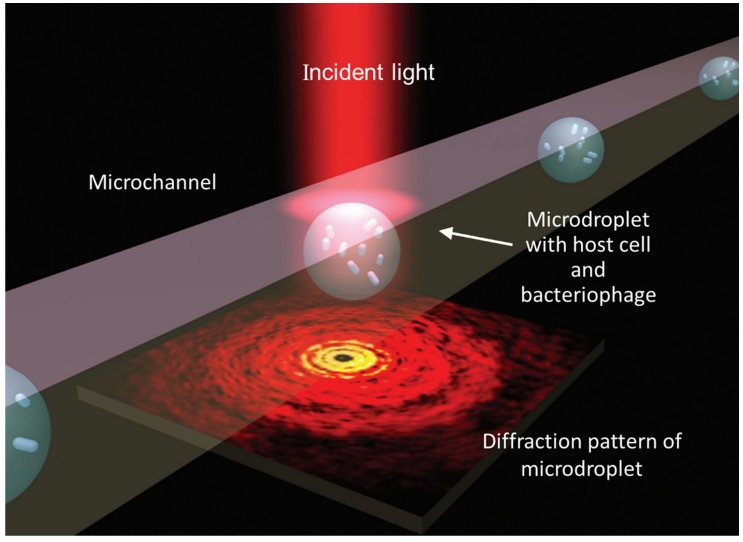
Schematic diagram of the optofluidic imaging based on microdroplets, co-cultured bacteriophage, and host cells (*E. coli*) [45].

Figure 5 shows the droplet optofluidic imaging system for phage monitoring, in which microdroplets were utilized as the reaction containers and light scattering was captured for optical imaging. When light is induced onto the droplets, including both the host cells and the phage, the light forms a scattering pattern on the image plane. The host cell growth rate was effectively correlated and the information can be taken from the photo-fluidic imaging signal. The optofluidic imaging system not only has high potential for monitoring water quality, especially for drinking water, but can also be used in clinical diagnosis and pathogenic bacteria detection in the food industry.

### 4.2. Bacteria Enrichment and PCR Detection

The enrichment and detection of *E. coli* O157:H7 in aqueous samples is also of significance. In Dharmasiri’s research [46], a microfluidic chip was fabricated to isolate and enrich *E. coli* O157:H7 cells with low abundance (<100 cells/mL) from water samples. The microchip could process eight kinds of samples independently or a sample pumped into eight different parallel microchannels to increase productive forces. After enrichment, cells were released and enumerated by utilizing benchtop real-time quantitative Polymerase Chain Reaction (PCR). The recovery of target cells from water samples was detected to be about ~72%, and the limit-of-detection reached six colony forming units (CFU) by utilizing slt1 gene as a reporter.

Lake and waste water samples can be analyzed with this device. Both the fabrication and operation processes were simple, which makes this kind of device attractive and competitive for the detection of pathogenic species from water samples, including bacterial pathogens with quite low frequencies.

### 4.3. Virus PCR Dection

An optofluidic quantitative PCR (MFQPCR) system was designed to detect 11 major human viral pathogens simultaneously [47]. The researchers collected samples from the Sapporo Sewage Treatment Plant in Japan to validate the presence of various viruses in the MFQPCR system. By using this system, the viral quantities obtained were nearly the same as in the traditional method. Thus, the direct and quantitative information of viral pathogens can be obtained from the MFQPCR system, which is used for risk assessments.

### 4.4. Automatic Microscopic Identification of Parasites

Optical microscopy is widely utilized as an analytical tool for biomedical and biological applications. In 2006, Heng et al. reported that the novel technique of optofluidic microscopy (OFM) [48] combines microfluidics and optics for low-cost microscopy imaging with high resolution. Instead of using lots of lenses and other optics of conventional optical microscopes, the OFM performs an imaging translation/scanning of the target biological specimen through a gold-plated array of holes at the bottom of the microchannels.

**Figure 6:**
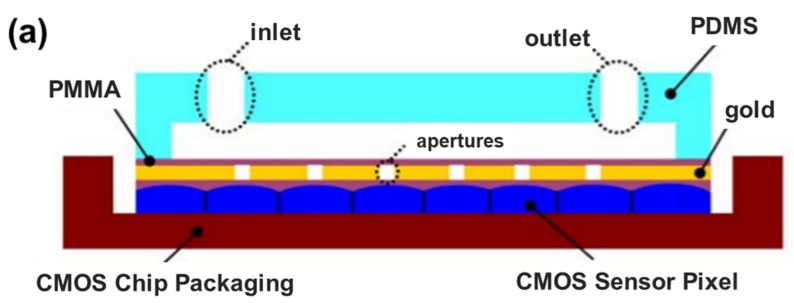

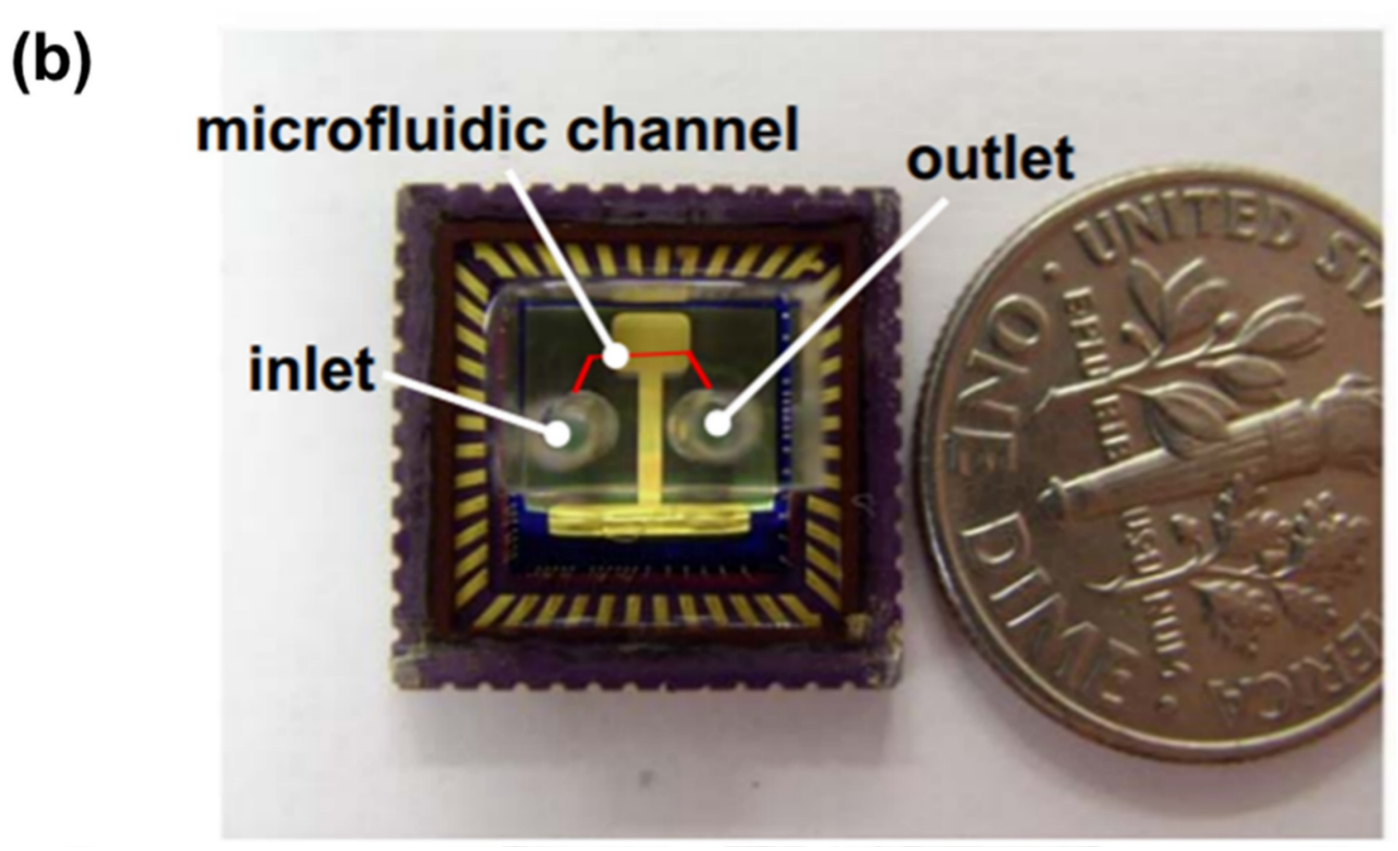
On chip OFM system for the detection of protozoan parasites in drinking water. Detection of disease-causing parasite species like *Caenorhabditis elegans* and *Giardia lamblia* was demonstrated using OFM technology. (**a**) Cross section view of the OFM device; (**b**) photo of the fabricated on-chip OFM device [49].

Lee et al. designed and set up a complete on-chip OFM system that was designed to image *Caenorhabditis elegans*, as shown in Figure 6 [49]. The OFM system can achieve high-resolution microscopic images beyond the sensor pixel size limitation by reconstruction of raw acquired images using a special image processing algorithm. OFM can also be used for imaging *Giardia lamblia* trophozoites and cysts, a parasite species causing infectious diseases and commonly found in poor-quality water. OFM has achieved a focal plane resolution of 800 nm. The sub-cellular morphology information is able to identify specific waterborne protozoan parasites. This study shows that the OFM technology can potentially be applied to develop autonomous, low-cost, and highly compact water analysis systems to monitor water quality in hazardous environments and underdeveloped countries.

## 5. Discussion and Outlook

As optofluidic devices for water quality monitoring develop rapidly and show high potential for applications, several national agencies have funded projects for the technology’s development and commercialization [50,51,52,53,54]. Many prototypes and quasi-ready products have been developed and validated in real-world applications. Autonomous lab-on-a-chip biosensor-based equipment has been prototyped and its online chemical detection performance validated on the coast of Sagrada of Greece [52]. A label-free detection system for rapid protozoa detection in drinking water has been prototyped and its online *Cryptosporidium* detection performance validated in Singapore [51]. Miyake’s group at Tokyo University demonstrated 3D-printing-based optofluidic analyzers for monitoring water quality, which have been adopted by Toshiba. More and more mature optofluidic devices have been applied in the water industry.

**Figure 7:**
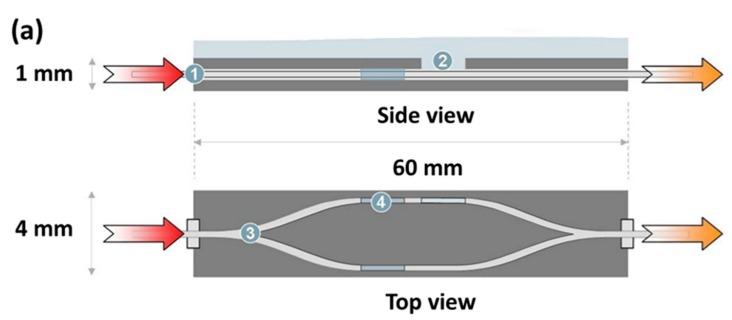

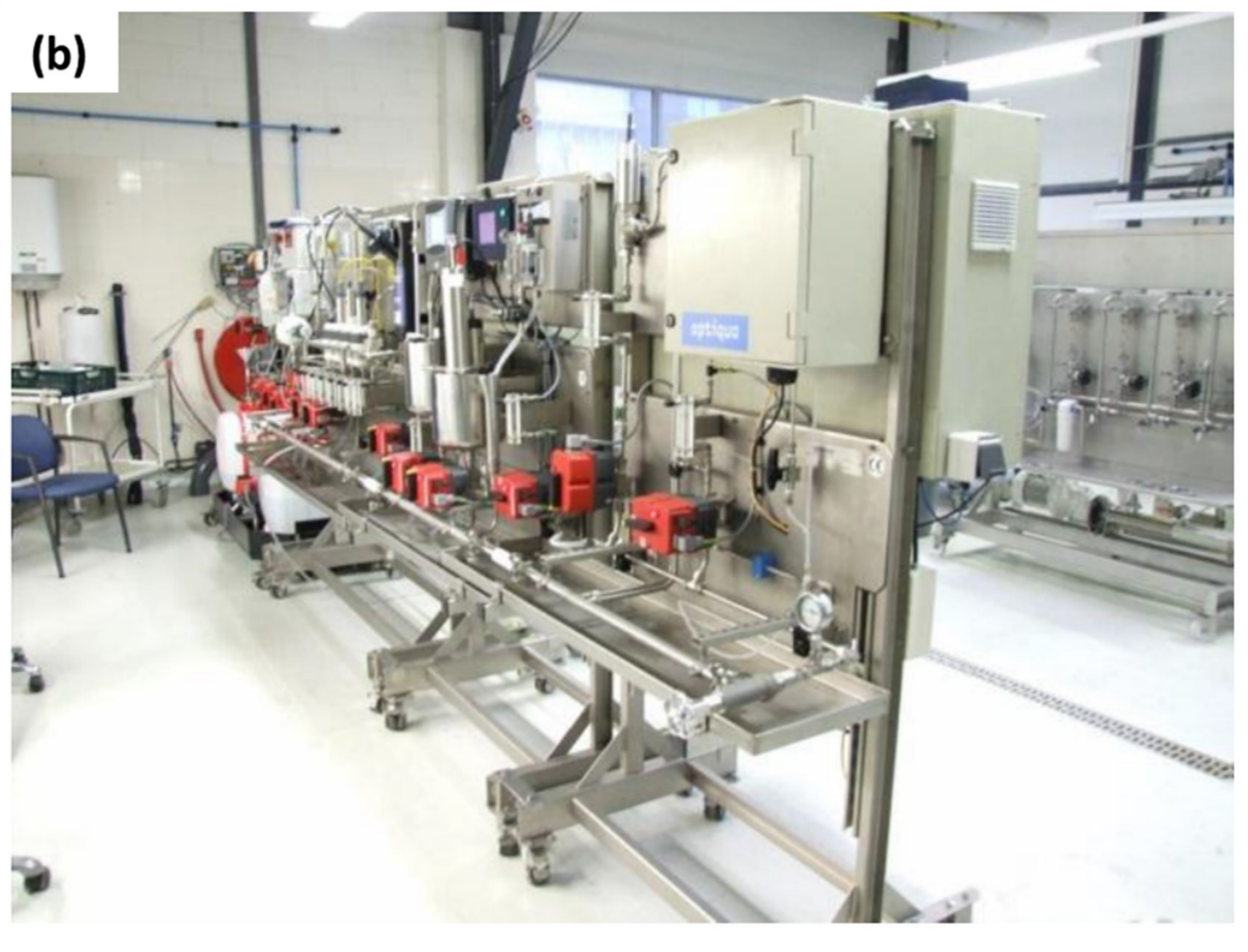
EventLab for online chemical event sensing. A new monitoring concept to continuously monitor the refractive index (RI) in real time is developed. RI is considered to be an indicator of the full spectrum of possible chemical contaminants and EventLab can be used as a highly sensitive (PPM level) generic optical sensor and an early-stage chemical contaminant warning system. (**a**) Schematic of the MZI chip used for measuring RI; (**b**) photograph of the optofluidic system installed in water plants for monitoring sodium chloride and so on [57].

Some companies have also successfully commercialized their optofluidic devices for online drinking water quality monitoring. Optiqua has commercialized Eventlab as a real-time water chemical event sensor to monitor drinking water network safety using an optofluidic based on the Mach–Zehnder interferometer, as shown in Figure 7a. Their products have been successfully applied by several utility companies worldwide [55,56]. Figure 7b shows a photograph of the optofluidic system installed in water plants for monitoring sodium chloride and so on.

Refractive index (RI) is used as an indicator to monitor the total chemical concentration and variance. A sudden RI change in the drinking water network will set off alarms for utility companies, prompting them to act immediately to prevent a second cross-contamination in the network. Their products have been successfully applied by several utility companies worldwide [57].

## 6. Conclusions

The application of optofluidics in water quality testing has become a mainstream trend in understanding water quality. Though impressive results and obvious significance have been reported, the promise of optofluidic systems for water industry will be realized only through continuous use of new technologies and converting these fundamental principles into prospective real-world applications. With the development of different types of optofluidic systems, real-time detection can be universal, and will make remarkable contributions to the water detection and environment research.
